# Combining the negative lymph nodes count with the ratio of positive and removed lymph nodes can better predict the postoperative survival in cervical cancer patients

**DOI:** 10.1186/1475-2867-13-6

**Published:** 2013-02-01

**Authors:** Ying Chen, Lei Zhang, Jing Tian, Xiubao Ren, Quan Hao

**Affiliations:** 1 Department of Gynecologic Oncology, Tianjin Medical University Cancer Institute and Hospital, Tianjin, 300060, China; 2 Department of Biotherapy, Tianjin Medical University Cancer Institute and Hospital, Tianjin, 300060, China; 3 Key Laboratory of Cancer Prevention and Therapy, Tianjin, 300060, China

**Keywords:** Cervical cancer, Lymph node, Pelvic lymphadenectomy, Prognosis

## Abstract

**Background:**

To evaluate the impacts of the negative lymph nodes (NLNs) count on the prognostic prediction of the ratio of positive and removed lymph nodes (RPL) in cervical cancer patients after radical hysterectomy and pelvic lymphadenectomy (RHPL).

**Methods:**

The positive and negative lymph node counts were calculated for 609 postoperative cervical cancer patients. The 5-year survival rate (5-YSR) was examined according to clinicopathologic variables. Cox regression was used to identify independent prognostic factors.

**Results:**

The NLNs count cutoffs were determined to be 10 and 25 with 5-YSR of 62.8% and 80.5%. The RPL of 13 patients who had the NLNs count of 10 or fewer was >20%. Among 242 patients who had 10 < NLNs count ≤ 25, 194 without positive nodes had the 5-YSR of 77.8%, 31 with 0% < RPL ≤ 5% had the 5-YSR of 3.2%, 15 with RPL > 20% had died when follow-up was completed. Among 354 patients who had NLNs count >25, 185 without positive nodes had the 5-YSR of 87.6%, 6 with 0% < RPL ≤ 5% had the 5-YSR of 25%, 15 with 5% < RPL ≤ 20% had the 5-YSR of 4.5%, and 2 with RPL >20% had died when follow-up was completed. Furthermore, stage, histologic grade and RPL were independently correlated with overall survival of cervical cancer patients after RHPL in the multivariate analysis.

**Conclusions:**

RPL was an independent prognostic factor. The NLNs count is a key factor for improvement of survival prediction of RPL in cervical cancer.

## Introduction

Cervical cancer is the third most common cancer and the fourth leading cause of cancer death in women worldwide, especially in developing countries [1]. Radical hysterectomy and pelvic lymphadenectomy (RHPL) are the standard treatment for FIGO (Federation International of Gynecology and Obstetrics) Stage IA_2_ to IIA_2_ cervical cancer, though nodal status does not affect the staging of cervical cancer [2]. The status of pelvic lymph node metastasis is one of the most important prognostic factors [3]. Masayoshi *et al*. reported the 5-year survival rate (5-YSR) of node-positive cervical cancer patients was 62.0%, which was significantly worse than that of node-negative patients (94.8%) [4].

Nevertheless, whether the more optimal prognosis can be achieved by increasing the number of dissected lymph nodes is still disputable. Some studies deemed that extensive lymphadenectomy increases survival. Pieterse *et al*. [5] reported that the disease-free survival (DFS) of node-positive patients with ≥18 removed lymph nodes was 20% higher than that of patients with <18 removed lymph nodes. Kim *et al*. [6] also demonstrated that removal of an increasing number of lymph nodes may be associated with better survival in patients with lymph node metastasis. However, Kim’s result was inconsistent with the study by Shah *et al*. [7], which revealed that more extensive lymphadenectomy was associated with improved survival in patients without nodal metastasis. Conversely, Prapaporn *et al*. [8] verified that there was not a significant improvement in DFS associated with removal of an increasing number of nodes regardless of nodal status. Furthermore, Soliman *et al*. emphasized that more extensive pelvic lymphadenectomies are associated with longer operating times, greater blood loss and postoperative complications [9].

However, for pelvic lymphadenectomy, the number of positive nodes is influenced by the surgical technique and the accuracy of the pathological examination. The ratio of positive and removed lymph nodes (RPL) may obviate possible confounding effects and be a more accurate representation of the status of pelvic lymph node metastasis [10]. Polterauer *et al*. [11] showed that RPL was an independent prognostic parameter in patients with lymph node-positive cervical cancer and superior to the number of positive lymph nodes in evaluation of overall survival. In contrast, Metindir *et al*. [10] reported that RPL did not reach statistical significance in their study, though this was likely due to the small number of patients with positive nodes (18 cases) and too small to have statistical power cutoffs in analysis. Because the lymph nodes examined in cervical cancer include positive and negative lymph nodes that are collected from patients simultaneously, the number of negative lymph nodes (NLNs) should be associated with the RPL and prognosis of cervical cancer after RHPL. However, to our knowledge, no data have been published that show a correlation between NLNs count and RPL or cervical cancer patient prognosis.

This study was conducted to elucidate the correlation between RPL, NLNs count and prognosis of cervical cancer patients after RHPL. We also sought to identify whether the NLNs count associated with RPL could preferably predict the survival of cervical cancer patients after RHPL.

## Results

### Cut point survival analysis for detecting the cutoffs of the RPL and NLNs count

The characteristics of 609 patients were listed in Table 1. The cut point analysis were performed to determine the best cutoffs of the RPL and the NLNs count to detect the greatest actuarial survival difference among the resulting subgroups. Differences among the subgroups were detected based on the magnitude of the log-rank statistic test. Results for the relevant cut points and stage subgroups according to the RPL and NLNs count are listed in Tables 2 and 3.

**Table 1:** Patients’ characteristics

**Characteristics**	**Media (range) or N (%)**
Media of age (years)	46 (23–68)
Media of follow-up (months)	67 (6 –90)
Pathologic type	
Squamous	506 (83.1)
Adenocarcinoma	74 (12.2)
Adenosquamous	29 (4.7)
Size of primary tumor	
≤4 cm	481 (79.0)
>4 cm	128 (21.0)
Histologic grade	
G_1_	61 (10.0)
G_2_	243 (39.9)
G_3_	305 (50.1)
Depth of primary tumor invasion	
≤1/2	355 (58.3)
>1/2	254 (41.7)
Stage (FIGO)	
IA2	95 (15.6)
IB1	317 (52.1)
IB2	73 (12.0)
IIA1	69 (11.3)
IIA2	55 (9.0)
Lymph-vascular space invasion	
No	518 (85.1)
Yes	91 (14.9)
Lymphadenectomy	
Pelvic	549 (90.1)
Pelvic + Periaortic	60 (9.9)
Neo-adjuvant chemotherapy	
Yes	128 (21.0)
No	481 (79.0)
Adjuvant therapy	
None	451 (74.1)
Concurrent chemoradiation	158 (25.9)
Lymph nodes metastasis	
No	516 (84.7)
Yes	93 (15.3)

**Table 2:** 1-, 3- and 5-year overall survival rates by RPL subgroup

**RPL subgroup (%)**	**Cases**	**1-YSR (%)**	**3-YSR (%)**	**5-YSR (%)**	***χ***^***2***^	***P***
0	516	92	85	83		
0 < RPL ≤ 5	10	90	50	18	31.508	0.000
5 < RPL ≤ 10	26	54	18	18	7.338	0.007
10 < RPL ≤ 15	11	36	8	0	0.056	0.813
15 < RPL ≤ 20	16	31	0	0	0.262	0.609
20 < RPL ≤ 30	9	22	0	0	3.965	0.046
>30	21	11	0	0	0.447	0.504

RPL: ratio of positive and removed lymph nodes; YSR: year survival rate.

**Table 3:** 1-, 3- and 5-year overall survival rates by NLNs count subgroup

**NLN subgroup**	**Cases**	**1-YSR (%)**	**3-YSR (%)**	**5-YSR (%)**	***χ***^***2***^	***P***
0	4	100	0	0		
0 < NLNs count ≤ 5	2	100	0	0	3.240	0.072
5 < NLNs count ≤ 10	7	71	0	0	0.293	0.588
10 < NLNs count ≤ 15	33	76	64	57	13.704	0.000
15 < NLNs count ≤ 20	95	74	36	58	0.027	0.869
20 < NLNs count ≤ 25	114	80	69	66	2.228	0.136
25 < NLNs count ≤ 30	146	90	85	81	8.466	0.004
30 < NLNs count ≤ 35	81	88	80	79	0.267	0.605
35 < NLNs count ≤ 40	54	89	83	78	0.016	0.901
NLNs count > 40	73	92	82	79	0.000	0.992

NLN: negative lymph node; YSR: year survival rate.

**Figure 1:**
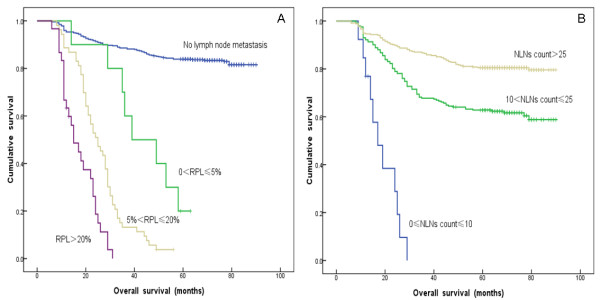
**A: Survival curve for 609 patients with cervical cancer after RHPL according to subgroups based on the ratio of positive and removed lymph nodes (RPL) (0%, 0% < RPL ≤ 5%, 5% < RPL ≤ 20% and >20%). B**: Survival curve for 609 patients with cervical cancer after RHPL according to subgroups based on negative lymph nodes (NLNs) count (0 ≤ NLNs count ≤ 10, 10 < NLNs count ≤ 25 and NLNs count > 25).

As shown in Table 2, the cutoffs for RPL required for verification of statistically significant survival differences between resulting subgroups were 0%, 5% and 20%. Using the Kaplan-Meier method to analyze overall survival (OS) rates according to these RPL subgroups, we observed significant differences in survival between all groups, including 516 patients with 0% RPL, 10 patients with 0% < RPL ≤ 5%, 53 patients with 5% < RPL ≤ 20% and 30 patients with RPL > 20% (*P*<0.001) (Figure 1A).

As shown in Table 3, the cutoffs for NLNs count required for verification of statistically significant survival differences between resulting subgroups were 10 and 25. Using the Kaplan-Meier method to analyze OS rates according to these subgroups, and the results showed there were significant survival differences among all groups, including 13 patients with 0 ≤ NLNs count ≤ 10, 242 patients with 10 < NLNs count ≤ 25 and 354 patients with NLNs count >25 (*P*<0.001) (Figure 1B).

### Univariate and multivariate survival analysis of 609 cervical cancer patients

Univariate analysis revealed that seven clinicopathological factors were significantly associated with OS of cervical cancer patients after RHPL: pathologic type, primary tumor size, histologic grade, depth of primary tumor invasion, FIGO stage, RPL and NLNs count (Table 4). Lower 5-YSR was more likely in patients with adenocarcinoma and adenosquamous carcinoma, primary tumor >4 cm, low differentiated, depth of primary tumor invasion >1/2, high RPL, and low NLNs count. Moreover, patients with cervical cancer of different substages (IA2 to IIA2) also exhibited statistically significant differences in overall survival. More specifically, patients with stage IIA2 disease had the lowest 5-YSR, while patients with stage IB1 disease had the highest 5-YSR among the five subgroups. Compared pairwise over strata, the 5-YSR of patients with stage IA1 and IB1 disease was not significantly different but others was obvious significantly different (Table 5).

**Table 4:** Clinicopathologic factors affecting the 5-YSR of cervical cancer patients

**Clinicopathologic characteristics**	**Cases**	**5-YSR (%)**	**χ**^**2**^	***P***
Age at diagnosis (years)			0.405	0.524
<46	266	83.0		
≥46	343	78.2		
Pathologic type			5.452	<0.001
squamous	506	88.5		
adenocarcinoma	74	58.6		
adenosquamous	29	48.3		
Size of primary tumor			235.681	<0.001
≤4 cm	481	87.5		
>4 cm	128	54.3		
Histologic grade			19.357	<0.001
G_1_	61	86.9		
G_2_	243	78.0		
G_3_	305	64.3		
Depth of primary tumor invasion			74.538	<0.001
≤1/2	355	84.5		
>1/2	254	77.5		
FIGO Stage			390.46	<0.001
IA2	95	86.3		
IB1	317	87.1		
IB2	73	47.9		
IIA1	69	63.6		
IIA2	55	0		
RPL (%)			457.556	<0.001
0	516	88.6		
0 < RPL ≤ 5	10	10.0		
5 < RPL ≤ 20	53	2.5		
RPL > 20	30	0		
NLNs count			92.372	<0.001
0 ≤ NLNs count ≤ 10	13	0		
10 < NLNs count ≤ 25	242	62.8		
NLNs count > 25	354	80.5		

RPL: ratio of positive and removed lymph nodes; NLNs: negative lymph nodes;

5-YSR: 5-year survival rate.

**Table 5:** Comparison of the median overall survival of patients with stage IA1 to IIA2 cervical cancer using Kaplan-Meier pairwise over strata analysis

**FIGO Stage**	**χ**^**2**^	***P***
IA2 *vs* IIA2	147.832	<0.001
IB1 *vs* IA2	0.000	0.996
IB2 *vs* IB1	77.868	<0.001
IIA1 *vs* IB2	6.103	0.013
IIA2 *vs* IIA1	81.266	<0.001

These seven variables were analyzed using the multivariate Cox proportional hazard model (Table 6). In that model, stage, histologic grade and RPL were the independent factors for evaluation of OS (P<0.001).

**Table 6:** Multivariate analysis of factors affecting the overall survival of 609 cervical cancer patients using Cox proportional hazard model

**Parameter**	***P***	**HR**	**95% CI**	
			**Lower**	**Upper**
Size of primary tumor	0.117	1.457	0.910	2.332
FIGO Stage	<0.001	1.793	1.510	2.127
Histologic grade	<0.001	1.696	1.314	2.189
Pathologic type	0.248	1.146	0.909	1.444
Depth of primary tumor invasion	0.954	1.012	0.675	1.516
NLNs count	0.132	1.260	0.932	1.703
RPL	<0.001	2.409	1.980	2.929

RPL: ratio of positive and removed lymph nodes; NLN: negative lymph nodes; HR: hazard ratio; CI: confidence interval.

### Correlation between RPL and NLNs count in predicting the survival of cervical cancer patients after RHPL

**Figure 2:**
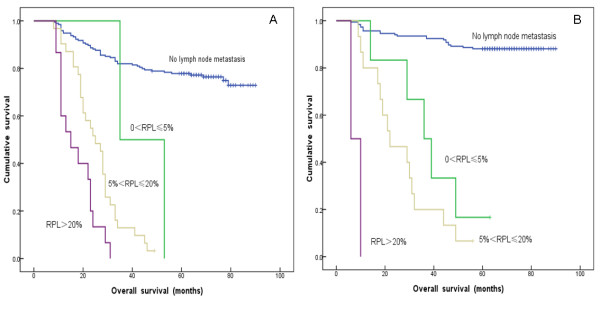
**A: Survival curve for 242 cervical cancer patients with 10<NLNs count ≤ 25 after RHPL according to subgroups based on the ratio of positive and removed lymph nodes (RPL) (0% < RPL ≤ 5%, 5% < RPL ≤ 20%, and >20%). B**: Survival curve for 354 cervical cancer patients with NLNs count >25 after RHPL according to subgroups based on the ratio of positive and removed lymph nodes (RPL) (0% < RPL ≤ 5%, 5% < RPL ≤ 20%, and >20%).

In our study, 13 patients with no more than 10 NLNs presented with >20% RPL. The media OS of these patients was 15 months, and all were dead when our follow-up was completed (Table 7). Furthermore, 242 patients had 10 < NLNs count ≤ 25. Among them, 194 patients with had a 5-YSR of 77.8%; 31 patients with 5% < RPL ≤ 20% had a 5-YSR of 3.2%; and 15 patients with RPL > 20% had all died when our follow-up was completed. Notably, two patients with 0% < RPL ≤ 5% had died when our follow-up was completed, but these results were not considered in the general evaluation because of the limited sample size. These observations indicated that the higher the RPL value, the lower the 5-YSR for patients with 10 < NLNs count ≤ 25 (*P* < 0.001) (Figure 2A, Table 7). In addition, 354 patients had NLNs count >25. Of these 354 patients, 185 without lymph node metastasis had a 5-YSR of 87.6%; 6 patients with 0 < RPL ≤ 5% had a 5-YSR of 25%; 15 patients with 5% < RPL ≤ 20% had a 5-YSR of 4.5%; and 2 patients with RPL >20% had died when our follow-up was completed. Thus, in this group of patients with high NLNs count, we also found that the 5-YSR significantly decreased as the RPL increased (*P* < 0.001) (Figure 2B, Table 7).

**Table 7:** Correlation between RPL and NLNs count in survival prediction of cervical cancer after RHPL

**RPL (%)**	**5-YSR for NLNs count of:**					
	**0 ≤ NLNs count ≤ 10**		**10 < NLNs count ≤ 25**		**NLNs count > 25**	
	**n**	**%**	**n**	**%**	**n**	**%**
0	-	-	194	77.8%	185	87.6%
0 < RPL ≤ 5	-	-	2	0	6	25%
5 < RPL ≤ 20	-	-	31	3.2%	15	4.5%
>20	13	0	15	0	2	0
5-YSR (%)	0		62.8%		80.5%	

RPL: ratio of positive and removed lymph nodes; NLN: negative lymph nodes;

5-YSR: 5-year survival rate.

From our results, we concluded that the 5-YSR for FIGO Stage IA_1_ to IIA_2_ cervical cancer patients increased with increasing NLNs count within specific RPL ranges (Table 7).

## Discussion

Systematic pelvic lymphadenectomy is an important part of surgical treatment for FIGO Stage IA_2_ to IIA_2_ cervical cancer. However, it remains an unresolved issue that a minimum number of nodes should be required to consider the lymphadenectomy as adequate. Prapaporn *et al*. suggested that more than 10 nodes should be removed for standard pelvic lymphadenectomy [8]. Similarly, removal of more than 11 pelvic lymph nodes was suggested as a quality indicator for pelvic lymphadenectomy in an EORTC-GCG study [12]. In this study, the number of pelvic lymph nodes removed ranged from 11 to 67, which meet the qualification of previous studies. Moreover, the status of regional lymph nodes is known to be an important indicator for the prognosis of cervical cancer patients [13].

Nevertheless, what remains controversial is if cervical cancer patients who have the same number of positive nodes but different numbers of removed pelvic nodes have similar overall survival. Several studies showed that positive nodal ratio was an independent prognostic parameter for OS of malignant solid tumors, including gastric cancer [14], colon cancer [15], esophageal cancer [16]. As for gynecologic malignant diseases, previous studies demonstrated the ratio of positive lymph node provides a significant prognostic value in epithelial ovarian cancer [17] and endometrial cancer [18,19]. However, Metindir *et al*. [10] and Polterauer *et al*. [11] drew diametrically opposite conclusions on the impact of RPL on the prognosis of cervical cancer patients. In this study, we selected cutoff values of 0%, 5% and 20% for further analysis. Survival analysis revealed that greater RPL values correlated with lower 5-YSR. Moreover, multivariate analysis showed that RPL was an important independent indicator for postoperative cervical cancer patients.

Recently, studies about NLNs associated with postoperative survival prediction for cancer patients caused more and more attention. NLNs dissection is a surrogate marker related to the quality of surgery; it reflects the extent of LN dissection. Previous studies had demonstrated the NLNs count is a marker of both the efficacy of lymph node evaluation and patient prognosis with some carcinoma. Schwarz *et al*. [20] reported that higher NLN counts were correlated with longer survival of gastric cancer patients after curative resection. Johnson *et al*. [21] showed that there was a marked decrease in disease-specific mortality as the number of negative nodes increased in patients with stage IIIB and IIIC colon cancer. In this study, we speculated that it is crucial for the total collected lymph nodes to comprise NLNs count, which is the basic guarantee for cervical cancer after RHPL. We identified that 5-YSR became higher as the NLNs count increased. This may be because NLNs have reduced potential for micro-metastasis. Theoretically, the higher the NLNs count, the more likely it is that resection will be optimal and not leave any potential metastasis behind.

Standard pathologic examination of lymph nodes is based on hematoxylin-eosin staining. Retrospective studies report that the incidence of micro-metastasis is between 1.5% and 15%, depending on the technique used to evaluate lymph node status [22]. The International Ludwig Breast Cancer group examined lymph nodes from 736 patients with negative lymph nodes and found an occult metastasis rate of 7% using serial sectioning alone and 20% using cytokeratin immunohistochemistry [23]. More importantly, they found that occult metastases detected by either method were associated with a significant decrease in disease-free and overall survival [22,23]. By using immunohistochemistry, Margrit *et al*. identified 8% of cervical cancer patients with lymph node micro-metastasis not initially identified by hematoxylin-eosin analysis [24]. Their findings are consistent with those of Lentz *et al*., who found micro-metastases in 14% of patients with stage IA1–IIA cervical cancer with histologically negative lymph nodes using anti-cytokeratin immunohistochemical staining [25]. Although we have not used immunohistochemical antibodies to detect micro-metastasis within NLNs, we demonstrated that increasing NLNs count could greatly improve the 5-YSR of cervical cancer patients. Therefore, we concluded that the higher the NLNs count, the higher the probability that micro-metastasis within lymph nodes will be found.

Additionally, we found advanced stage, low histologic grade and high RPL were independent markers of poor prgnosis for cervical cancer patients, which partially was consistent with previous study [26].

## Conclusions

In this study, we show that the 5-YSR of postoperative cervical cancer patients increased as the NLNs count increased for specific ranges of RPL. The prognostic prediction of the RPL for cervical cancer after RHPL would thus be greatly improved if combining the NLNs count. Conclusively, RPL was an important independent prognostic factor, and the NLNs count was a key factor for improvement of survival prediction by RPL in postoperative cervical cancer patients.

## Methods

### Patients and treatment

A total of 718 women with cervical malignant carcinoma underwent RHPL at the Department of Gynecologic Oncology, Tianjin Medical University Cancer Institute and Hospital from January 1998 to December 2006. Patients with special pathological cancer and without integrated follow-up were excluded. A total of 609 patients were included in this study. Approval by the Institutional Review Board of Tianjin Medical University Cancer Institute and Hospital was obtained in advance, and the informed consent requirement was waived because the current study was performed by retrospective review. In addition, because the stage was updated in 2009, patients originally staged at IIA were divided into IIA_1_ and IIA_2_ according to the initial examination description in the medical records. None of the enrolled had underlying disease that would influence survival.

Patients with primary tumors >4 cm received 1 to 3 cycles of cisplatin-based neo-adjuvant chemotherapy. For adjuvant treatment after RHPL, intermediate and high risk factors were evaluated by histological examination. Intermediate risk factors included large tumor size (>4 cm), deep stromal invasion (>1/2) and lymph-vascular space invasion, whereas high risk factors were positive resection margin, parametrial invasion and lymph node metastasis. The criteria for adjuvant treatment after surgery were ≥2 intermediate or ≥1 high risk factors. Concurrent chemoradiation was conducted by 2 to 3 cycles of platinum-based chemotherapy and whole pelvic irradiation (50 Gy/25 fr) as postsurgical adjuvant treatment. After RHPL surgery, all patients were followed up every 3 months for 2 years, then 6 months or until death.

Tissue samples were sent to the department of pathology for histological examination. Board-approved pathologists, specialized in gynecological pathology, assessed the pathological specimens. Formalin fixed samples from each defined topographical localization were described macroscopically for size, consistency, and number of lymph nodes. To guarantee detection of all the nodes, even small nodes, remaining fatty tissue was also embedded. Finally, paraffin blocks were serially sectioned and stained with haematoxylin and eosin for microscopical examination.

### Statistical analysis

The RPL and NLNs count were categorized by cutoffs determined using cut point survival analysis [27]. The survival rate was calculated using the Kaplan-Meier method, and the log-rank test was used to compare survival curves. Factors that were deemed of potential importance by univariate analysis were included in multivariate analysis. Multivariate analysis of overall survival was performed using the Cox proportional hazard model for variable selection. A result was considered significant when the *P* value was <0.05. All statistical analysis was performed with the SPSS statistical analysis program package, version 17.0 (SPSS, Chicago, IL).

## Abbreviations

RHPL: Radical hysterectomy and pelvic lymphadenectomy; RPL: Ratio of positive and removed lymph nodes; NLN: Negative lymph node; OS: Overall survival; 5-YSR: 5-Year survival rate.

## Competing interests

The authors declare that they have no competing interests.

## Authors’ contribution

HQ designed the experiments. CY, ZL, and TJ summarized the data. CY, ZL, RXB, and HQ analyzed the data. CY wrote the paper. All authors have read and approved the final manuscript.
